# Exploring the Complexity of Intellectual Disability in Fetal Alcohol Spectrum Disorders

**DOI:** 10.3389/fped.2014.00090

**Published:** 2014-08-26

**Authors:** Aniruddho Chokroborty-Hoque, Bonnie Alberry, Shiva M. Singh

**Affiliations:** ^1^Molecular Genetics Unit, Department of Biology, University of Western Ontario, London, ON, Canada

**Keywords:** neurodevelopment, fetal alcohol spectrum disorders, mouse models, epigenetics, stress, environmental enrichment, intellectual disability, gene expression

## Abstract

Brain development in mammals is long lasting. It begins early during embryonic growth and is finalized in early adulthood. This progression represents a delicate choreography of molecular, cellular, and physiological processes initiated and directed by the fetal genotype in close interaction with environment. Not surprisingly, most aberrations in brain functioning including intellectual disability (ID) are attributed to either gene(s), or environment or the interaction of the two. The ensuing complexity has made the assessment of this choreography, ever challenging. A model to assess this complexity has used a mouse model (C57BL/6J or B6) that is subjected to prenatal alcohol exposure. The resulting pups show learning and memory deficits similar to patients with fetal alcohol spectrum disorder (FASD), which is associated with life-long changes in gene expression. Interestingly, this change in gene expression underlies epigenetic processes including DNA methylation and miRNAs. This paradigm is applicable to ethanol exposure at different developmental times (binge at trimesters 1, 2, and 3 as well as continuous preference drinking (70%) of 10% alcohol by B6 females during pregnancy). The exposure leads to life-long changes in neural epigenetic marks, gene expression, and a variety of defects in neurodevelopment and CNS function. We argue that this cascade may be reversed postnatally via drugs, chemicals, and environment including maternal care. Such conclusions are supported by two sets of results. First, antipsychotic drugs that are used to treat ID including psychosis function via changes in DNA methylation, a major epigenetic mark. Second, post-natal environment may improve (with enriched environments) or worsen (with negative and maternal separation stress) the cognitive ability of pups that were prenatally exposed to ethanol as well as their matched controls. In this review, we will discuss operational epigenetic mechanisms involved in the development of intellectual ability/disability in response to alcohol during prenatal or post-natal development. In doing so, we will explore the potential of epigenetic manipulation in the treatment of FASD and related disorders implicated in ID.

## Intellectual Disability

Mental retardation is a highly diverse group of cognitive disorders. The fifth edition of the *Diagnostic and Statistical Manual of Mental Disorders* (*5th ed*.; *DSM*-*5*) characterizes mental retardation [revised as Intellectual Disability (ID) in the fifth edition) by impairments of general mental abilities that fall under the conceptual, social, and practical domains of adaptive functioning. Individuals with ID have intelligence quotient (IQ) scores below 70, approximately two standard deviations or more below the population average score (1). They represent 1–3% of the population in Canada, and males are affected more often than females (2). Roughly two-thirds of ID individuals have mild-to-moderate impairments while the remaining third are severely affected (2). In 2006, the Participation and Activity Limitation Survey by Statistics Canada, found the largest proportion of ID occurs in the 15–24 age group (29.3%) (2). The survey also found that compared to individuals with physical disabilities (52.7%), people with intellectual disabilities are far less likely to be employed (26.1%) with an inverse trend of socio-economic status with respect to prevalence (2). In America, the lifetime costs are expected to be near $50 billion for individuals born in 2000 with ID (3).

Intellectual disability and its variable manifestations are often attributed to aberrations in neurodevelopment that are complex, poorly understood, and long lasting in mammals. It begins early during embryonic development but may take years to complete and is finalized in early adulthood. Further, it involves a delicate choreography of cellular, molecular, and physiological processes directed by the fetal genotype in close interaction with the environment at every step, over time. Consequently, it covers periods before birth, during birth, and/or the childhood years. The causes of ID are complex and multifactorial. In some rare cases, the primary determinants of ID are known. For example, rare chromosome number defects [Down syndrome (4, 5)], inherited chromosomal disorders [Fragile X syndrome (6)], and a number of single gene mutations (7–9) are known to cause a spectrum of intellectual. Unfortunately, ID tends to be heterogeneous with a wide spectrum of manifestations. Additionally, neurodevelopmental exposure to a variety of drugs and chemicals can result in ID, such as cocaine, alcohol, and lead, among others (10). ID is not a single disorder, rather the result of a plethora of causations involving both genes and environment. The understanding of the developmental processes associated with ID and related abnormalities calls for a research focus on specific diagnosis potentially caused by a single known factor, an experimental model that is easier to manipulate and interpret. In this discussion, we will use fetal alcohol spectrum disorders (FASD) as a case study of the complexity of ID.

## Fetal Alcohol Spectrum Disorders

Fetal alcohol spectrum disorder with all its manifestations results from a single initial cause, prenatal alcohol exposure (PAE). It includes mild behavioral and learning impairments, to the most severe form called Fetal Alcohol Syndrome (FAS). FAS may include ID as well as birth defects (11). The intellectual deficits in FAS and FASD are highly variable and heterogeneous. These symptoms are considered chronic, often co-occurring with other mental impairments, and manifesting during the developmental period. FASD represents one of the most common causes of learning disabilities, cognitive deficits, and ID (12). The severity of impairments is evaluated using both clinical assessments and standardized testing of intelligence.

Obtaining a diagnosis of FASD requires input from various medical professionals, with estimated costs associated with the diagnostic procedure up to $7.3 million per year in Canada (13). Unfortunately, despite increasing public education on the dangers of PAE, the occurrence of alcohol exposed pregnancies remains a significant societal problem. In Canada, 74.4% of women surveys reported alcohol use in the past year (14). Most disappointing is the prevalence of PAE in certain high-risk groups. In Fort McMurray, Alberta, almost 50% of pregnant women surveyed reported consuming an alcoholic beverage since learning of their pregnancy (15). In a survey of women in Arctic Quebec, over 60% of women reported alcohol consumption during pregnancy (16). While the rates of PAE are often considered high, not every reported incident of alcohol exposure results in FASD. In Canada, the more severe FAS is estimated to occur at rates of 1–2 per 1000 live births (17), while the more mild FASD occurs much more often, at a rate of 9 per 1000 (18). In northeastern Manitoba, estimates of FASD incidence are as high as 14.8 per 1000 births (19). Children entering child care systems, such as foster care and orphanages, also represent a subpopulation with higher incidence of FASD, with estimates at 60 per 1000 children (20). A 2010 study found that 48% of pregnancies in the United States were unintended (21), and over 30% of women reported consuming alcohol while pregnant (22). The Centre for Disease Control and Prevention reports FAS rates in the USA ranging from 0.2 to 1.5 per 1000 live births solidifying the position of FAS as one of the leading preventable causes of intellectual disabilities (23).

How neurodevelopmental alcohol exposure may cause ID is poorly understood. It is a critical area of research. Such studies are not always feasible in humans. In this review, we will present arguments to suggest that studies on the mechanisms in the development of intellectual disabilities could be modeled in suitable animal models using PAE. It allows coverage of prenatal as well as post-natal development. Specifically, we will focus on behavioral data to show that B6 mice offer an opportunity to assess the effect of neurodevelopmental time specific PAE on molecular processes that are affected by alcohol and may lead to the manifestation of ID and related abnormalities. Additionally, it allows controlled post-natal manipulation (negative stress or positive enrichment) on the manifestation of mental deficits in pups generated with and without PAE.

## Mouse Model of FASD Research

It is understandable that most research on the mechanisms involved in the development of FASD has concentrated on animal models, particularly mice (24–27). To this end, our laboratory has established two forms of neurodevelopmental *in vivo* alcohol treatment in B6 mice. The first uses injections at any time during neurodevelopment on time-mated females and the second uses free choice of 10% alcohol or water as the source of liquid for pregnant females. The pregnant B6 mothers prefer (~70%) to drink a 10% ethanol in water solution over water. The two methods equate to the two forms of PAE in humans; binge (injection) at any time during pregnancy and continuous maternal drinking (preference) during pregnancy. The resultant pups from the two treatments show alcohol specific phenotypes; developmental delays, increased anxiety, learning deficits, and pronounced deficits in visuo-spatial memory (27–29). They also exhibit delayed neural reflexes, aberrant limbic coordination, elevated levels of anxiety, and spatial-memory deficits (27). To better ascertain the effects of ethanol on critical neurodevelopmental time points, we have mimicked binge-like drinking episodes at critical times, representing equivalents to the three trimesters in humans. The trimester three equivalent represents a “brain growth spurt” – dominated by synaptogenesis during the first 2 weeks in B6 newborn pups (29, 30). It is a period marked by the formation of extensive neural connections that form the basis for much of the cell-to-cell communication in the brain. The ability of ethanol to trigger widespread neurodegeneration during synaptogenesis is accompanied by the upregulation of stress-related and apoptosis-related genes and a down-regulation of genes related to protein synthesis, mitosis, synaptic formation, and maintenance (28, 30, 31). The third trimester equivalent ethanol exposure also results in increased anxiety-like behavioral traits and pronounced recognition memory and visuo-spatial memory defects (29). The results show that most PAE treatments in B6 mice cause developmental as well as behavioral deficiencies that are compatible with manifestations of FASD. Additional studies regarding timing of ethanol exposure have found exposure during the first trimester equivalent leads to decreases in cerebellar volume, while second trimester equivalent exposure leads to decreased hippocampal volume (32). The model also allows further studies on specific brain regions that may offer novel insights. The hippocampus is one of the brain regions that may be important in the understanding of the complexity of FASD phenotypes. The primary role of the hippocampus is memory consolidation (33), emphasized by hippocampal lesions leading to impaired spatial learning in mice (34). PAE leads to learning and memory deficits via changes in the hippocampus (35). Ultimately, some of the behavioral effects of PAE may be a result of molecular changes in the hippocampus. The molecular effects of PAE have been well characterized using animal models under a plethora of conditions (various neurodevelopmental stages and different dosages of alcohol) and all of them have shown that PAE affects epigenetic and genetic processes and various neurodevelopmental pathways (36–38). A single (or in most cases, multiple) instance of alcohol exposure during fetal development can result in a lifetime of behavioral and cognitive deficits. Such results show that PAE treatments in B6 mice cause deficiencies that are comparable to the manifestations of FASD in humans.

## Molecular Etiology of FASD: Gene Expression and Epigenetic Marks

The development of genomic technologies has allowed the search for molecular mechanisms underlying deficits following PAE. In both cultured neurons and *in vivo* evidence, ethanol has been shown to induce programed cell death 4 (PDCD4) protein synthesis, ultimately resulting in neuronal growth abnormalities in a rat model of PAE (39). Ethanol has also been shown to induce apoptosis via ceramide pathways, alongside stress-related kinases during development in cultured rat astrocytes (40). Direct treatment of ethanol on cultured neural stem cells often results in a host of changes at the level of gene expression. It includes *Dnmt1, Uhrf1, Ehmt1, Ash2 l, Wdr5*, and *Kdm1b* transcripts that have been shown to have significantly different levels of gene expression following ethanol exposure *in vitro* (41).

We have attempted such studies on B6 *in vivo* (42). The results show that multiple ethanol-treatment paradigms that result in FASD phenotypes also show changes in gene expression (28, 30). Such changes occur with respect to neurodevelopmental timing of exposure. More important they are representative of genomic alterations that are dependent on the biological processes occurring at the time of ethanol exposure (30, 42). Interestingly, ethanol exposure initiates alterations in a set of genes (short-term effect) that primarily affect cellular compromise and apoptosis representative of ethanol’s toxic effects. In the long term, however, genes affected following PAE are very different and involve various cellular functions including epigenetic processes such as DNA methylation, histone modifications, and non-coding RNA regulation that may underlie long-term changes to gene expression patterns (43). These may be initiated by ethanol-induced alterations to DNA and histone methylation, particularly in imprinted regions of the genome, affecting transcription, which is further fine-tuned by altered microRNA (44). These processes are likely complex, genome-wide, and interrelated. The epigenetic changes may be responsible for the FASD-related alterations in gene expression. Additionally, the epigenetic changes acquired may remain stable for life and maintain the manifestation of FASD.

At least two features of this system are encouraging and offer hope for people affected with FASD and related disorders. First, human brain development is not complete at birth, rather it continues for decades. More important, the neurodevelopment during this period is rather malleable and responsive to post-natal environment. Consequently, it may provide an opportunity to direct/maneuver post-natal brain development and alter the course of development of FASD and related endophenotypes. Second, the underlying epigenetic changes brought about by PAE represent an adjustable process. Specifically, DNA methylation is known to be reversible, and may be altered using different strategies. This promise and hope offered by the two features (continuity of brain development after birth and potential to change PAE epigenetic marks) have remained poorly explored in FASD-related studies. We will present preliminary results to argue that the continuum of post-natal neurodevelopment offers an opportunity to ameliorate the effect of prenatal alcohol and adjust/restore the final outcome.

## Post-Natal Environment Neurodevelopment and Functioning

Mammalian neurodevelopment is a long-lasting continuum. It begins early and finalized in early adulthood. It is also closely orchestrated and sensitive to prenatal as well as post-natal environment, particularly stresses. It makes it nimble with potential to incorporate desirable post-natal experiences. The mechanism behind this potential although recognized is not fully understood. What is known is that post-natal processes contribute to the life-long changes in behaviors and mental abilities. Also, it may result via responsiveness of the hypothalamic–pituitary–adrenal (HPA) axis (45), the primary physiological regulator of the environmental stress in mammals. Perhaps, the strongest evidence for this effect comes from post-natal handling of rodents. It involves daily separation of pups from the mother (3–15 min), a stressful event, for the first few weeks of life. Such pups show decreased stress reactivity in adulthood (46–48). Also, pups exposed to extended positive maternal care show decreased fearfulness and more modest HPA responses to stress (49, 50). Similar results have also been reported in non-human primates (51), and humans (52, 53). In each case, variations in post-natal conditions promote hippocampal synaptogenesis and spatial learning and memory through systems known to mediate experience-dependent neural development (54). The question of how post-natal environment causes such a dramatic effect in mammals has formed a fruitful area of research in recent years. It argues that this effect may be realized via the effectiveness of HPA axis.

An underlying mechanism behind the effect of post-natal environment is provided by studies by Michael Meaney and his collaborators. They found that increased licking and nursing by rat mothers altered DNA methylation of hippocampal glucocorticoid receptor in the pups. Further, the altered methylation is directly related to the development of HPA responses to stresses through tissue specific effects on gene expression (55). The results also emphasize that there is a critical period for such effects to be realized. It is particularly effective in early post-natal periods. This relationship between maternal care and gene expression via DNA methylation argues for environmental reprograming that is stable and may form the basis for the developmental origin of vulnerability to defects (56). These results have now been replicated in a number of mammals including humans. They argue that early life events can alter the methylation (epigenetic) state of relevant genomic regions, the expression of which may contribute to individual differences in the risk for pathology and diseases of fetal origin (57). Given this understanding, it is natural to consider post-natal enrichment in correction of any epigenetic pathology. It is particularly relevant in cases of FASD that are caused by alcohol-induced alterations in DNA methylation. In fact, one may postulate potential involvement of DNA methylation (58) at every step in neurodevelopment including responses to environment prenatally as well as postnatally.

**Figure 1:**
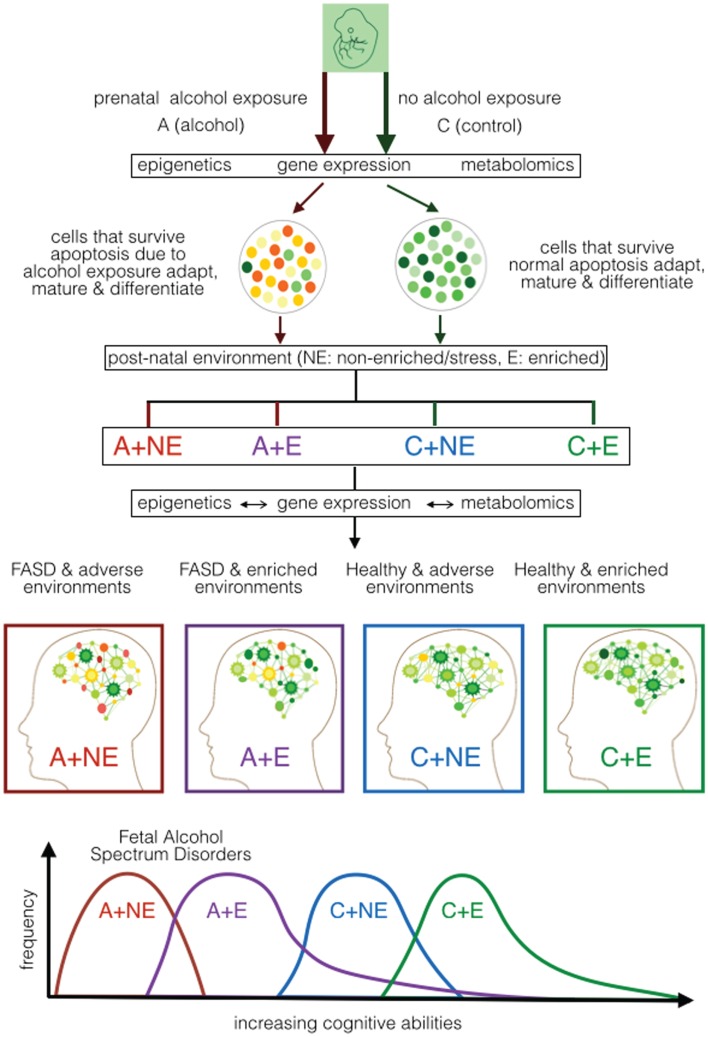
**Proposed developmental model of cognitive ability in FASD based on mouse models of prenatal alcohol exposure**. It shows the effect of prenatal alcohol (A) or control (C) under normal/non-enriched (NE) and enriched (E) conditions during early post-natal development. It portrays the effect of prenatal alcohol on epigenetic, gene expression, and metabolomic alterations during neurodevelopment as well as the effect of post-natal conditions on epigenetic, gene expression, and metabolomics during post-natal development. The expected cognitive ability of the mice subjected to the four treatment combinations (A–NE, A–E, C–NE, and C–E) is projected. It suggests that the post-natal environment is capable of ameliorating (at least partly) the effect of prenatal alcohol and other effects that may affect mental ability of the newborn.

The results available have allowed us to propose alterations in the sequential continuum of neurodevelopment in FASD over a longer time frame – from fertilization to maturity (Figure 1). It shows the continuum of neural development with and without prenatal alcohol that result in metabolomic changes leading to either FASD or not. It recognizes that the manifestation of this outcome is not fixed. It must follow additional development and refinement in a given post-natal environment. Once again, post-natal environment may affect the developing brain via epigenetic and metabolomic alterations. We argue that such alterations will vary and depend on the nature (heavily enriched to heavily stressful) of the post-natal environment. Consequently, the effect of post-natal environment may permit recovery from prenatal effects [enriched environments (EE)] or add additional defects (stressful environment). The model covers molecular processes that underlie the initiation, progression, and completion of neurodevelopment and any role prenatal or post-natal stress may have during gestation, birth, and post-natal development. The model recognizes that the effect of post-natal environment is not restricted to cases with PAE. Rather, it is expected to have an impact on cases where there is no exposure to alcohol. Further, although the nature of prenatal stress is well defined, the nature of post-natal environment that will have a positive and negative effect remains rather generic and needs to be carefully investigated. We will discuss this model further using ongoing experiments.

## Assessing the Post-Natal Environment on FASD

Fetuses exposed to alcohol prenatally have poor growth in the womb. Consequently, they are born with low birth weight (59). Further, almost none of such babies have normal brain development. They also show decreased muscle tone, poor coordination, and slow growth rate (59). Naturally, newborns with FASD are dependent on post-natal care by the mother for their future development. Also, they are often born in suboptimal families and raised in suboptimal conditions, particularly in the previously outlined high-risk subpopulations. Consequently, an unfavorable post-natal environment often provides a continuation of prenatal developmental insults thereby increasing the risk and severity of the PAE outcome. It has been argued that an enriched post-natal environment may have an ameliorating effect on the brain development in the FASD babies but the effect of post-natal environment on the development of FASD phenotypes has not been adequately explored. We will assess the results of stressful and enriched post-natal environment on the growth, development, and mental ability of newborns with diagnosed with FASD.

### Stressful post-natal environment in FASD

Results available in the literature argue that the combined effect of PAE and post-natal stress worsen the behavioral and structural effects of alcohol exposure alone (60, 61). Further accumulative stressors over time may contribute to increased risk of depression in FASD via HPA axis dysregulation (62). In humans, there are sex-differences in stress regulation, in that females show greater changes in heart rate, while males exhibit more alterations in cortisol levels (63). In primates, the combination of PAE and maternal stress led to a reduction in birth weight in males, but not females – further highlighting the sex-specificity. Additionally, both sexes show HPA axis responses following maternal separation stress (64). In rodent models of PAE, the basal levels of corticosterone and adrenocorticotropin (stress-related molecules) are unaltered, but subjects are hyper-responsive to stressors in adulthood (65). Chronic stress leads to increases in corticosterone in ethanol exposed females following an acute stress event (66), and prolonged exposure to stressors in ethanol exposed males leads to overactive HPA response (65). In B6 mice, maternal separation stress on normal pups is often used to model chronic early life stress. It uses 3 h of separation per day from post-natal days 2–14 that can result in anxiety-like behaviors in adult mice (67). The resulting mice display increased anxiety-like behaviors on open-field testing (68) similar to those observed in PAE models without maternal separation. Interestingly, behavioral abnormalities including learning and memory deficits induced by PAE and prenatal stress may be moderated by administration of BDNF (69). Further, such effects may be due to changes in hippocampal gene expression (70, 71). The general conclusion is that stressful post-natal environment may add deterioration on young’s exposed to prenatal alcohols. The specific interaction between prenatal alcohol and stressful post-natal environment however, has not been sufficiently examined.

### Post-natal environmental enrichment in FASD

Prenatal alcohol causes FASD. Also, how prenatal alcohol may manifest the development of FASD is becoming apparent. One of the next logical questions in FASD research deals with the role of post-natal environment. Most FASD children are born into an environment of malnutrition and drug and nicotine abuse (72, 73). In addition, poor socio-economic lifestyles along-with neglectful parenting, exacerbate the behavioral and cognitive abnormalities so characteristic of FASD children. It has been hypothesized that an enriched post-natal environment may lessen the severity of the manifestation in a newborn diagnosed with FASD. An enriched post-natal environment may involve intensive physical, cognitive, and behaviorally challenging environments (74, 75). The repeated exposure to counseling sessions and specialized classes with an aim to develop verbal, math, and social skills helps ameliorate some, if not all behavioral and cognitive deficits. While some interventions manage to lessen stress and anxiety levels in FASD children, cognitive disabilities still remain at large. However, such rehabilitative therapies have been unsuccessful in improving the spectrum of ID in FASD. What is needed is a better understanding of the molecular events that follow rehabilitative therapies in humans. To this end, it will be desirable to answer the question: Do rehabilitative therapies target the very same affected molecular pathways that cause FASD or do they have different molecular mechanisms? Such questions are better explored using animal models.

What constitutes “rehabilitative therapies” in rodent models of FASD? Ethanol exposed rats and mice that are subjected to physically and cognitively challenging environments (EE) tend to be less stressed and have improved memory performance (76). Given how fetal alcohol exposure affects neurodevelopment, it is possible that the effectiveness of EE result from a targeted activation of specific molecular mechanisms that modify brain structure and function and are ultimately expressed as “rehabilitated” behaviors. Compared to standard housing conditions (non-enriched) with shoe-box sized cages and basic food and housing, enriched cages tend to be much larger. The latter have toys of various shapes, sizes and textures, tunnels, nesting material, heavy bedding, and access to running wheels and ladders. The objects and their locations are changed weekly. Such environments facilitate mice to burrow, climb, chew, run, and explore new objects and placements, thereby engaging and developing cognitive processes. To eliminate stress due to isolation and or lack of social interaction, all mice, whether in standard or enriched cages are socially housed.

Our lab, amongst others has been interested in learning more about the effects of a positive, enriched post-natal environment on mice exposed to alcohol prenatally. Our first objective has been to demonstrate that environmental enrichment can ameliorate some, if not all of the behavioral and cognitive deficits that are characteristic FASD phenotypes. Four groups of mice have been generated: Control/Saline (C) mice living in enriched (CE) and non-enriched (CNE) conditions and prenatal alcohol exposed mice (A) living in enriched (AE) and non-enriched (ANE) conditions. Our results show that FASD mice that have been exposed to environmental enrichment (i) exhibit a fewer number of anxiety-like traits (as evidenced by more time spent in the light-region of the light–dark box and open-arms of the elevated-plus maze) and (ii) perform relatively better in learning and memory tests (as evaluated by the novel-object recognition and the Barnes maze). This experimental design has also allowed us to establish that enrichment not only ameliorates behavioral and cognitive deficits of affected mice (AE versus ANE) but improves these characteristics in normal, healthy control mice that had never been exposed to alcohol (CE versus CNE). Group comparison has also shown that prenatal ethanol exposure causes permanent and long-lasting damage to the developing brain. Further, the post-natal environmental enrichment is successful in ameliorating these deficits only to a certain extent. The mechanism involved in this amelioration is poorly understood and deserves further research.

The long-lasting effects of environmental enrichment have implicated changes in epigenetic machinery. Such results in conjunction with other lines of evidence show that the DNA methyltransferases (DNMTs) and histone acetyltransferases (HATs) are essential in neurodevelopment activities such as neural stem cell proliferation, differentiation, and synaptic plasticity (77–79). Work by Rampon et al. was among the first to show that DNMTs are preferentially up-regulated in the brains of healthy mice that have undergone environmental enrichment (80). While a number of genes involved in neuronal structure, neural plasticity, and synaptic signaling were up-regulated, the highest levels of induction was found in DNMTs. These enzymes are critical in neural cell differentiation induced by nerve growth factors (80). In 2011, Lopez-Atalaya et al. investigated the role of the histone acetyltransferase CREB-binding protein (CBP) in the context of environmental enrichment (81). CBP has been shown to be involved in neural plasticity and memory processes in the brain. Dysregulation of CBP is associated with a complex epigenetic disorder known as Rubinstein–Taybi syndrome, characterized by behavioral and cognitive deficits. CBP-deficient mice undergoing environmental enrichment have ameliorated physiological and behavioral deficits. In addition, multiple roles of CBP in neurogenesis and neuroadaptation to environmental changes were identified (81). Environmental enrichment has been shown to cause a dramatic increase in IDNA levels of BDNF, with concomitant widespread changes in histone methylation at various BDNF promoters and no change in the expression levels of several brain-specific microRNAs (82). Various other studies have pointed out the important role of BDNF in learning and memory processes (83, 84), particularly how BDNF shapes the cognitive and stress-response trajectory of neurodevelopment through interactions with the HPA axis (85–88). Our lab and others are currently investigating the effects of environmental enrichment on mice following alcohol exposure in the context of BDNF and its associated epigenetic marks to gain a better understanding of how the post-natal environment acts to ameliorate negative phenotypic outcomes as a result of alcohol exposure.

## Synthesis and Future Perspective

Most intellectual disabilities in children are caused by neurodevelopmental aberrations. Often they involve complex interactions of genes and environment over prenatal and post-natal periods. For example, intellectual disabilities in FASD are caused by PAE that disrupts neurodevelopment via alterations in gene expression. This affects a number of pathways that undergo changes during ontogeny over time. Here, the primary effect of alcohol covers cellular compromise and apoptosis, the expected toxic effect of ethanol. It leaves a molecular footprint that is shared among neurological disorders. The genes affected are related via hub molecules. More important, these results may last for life. We attribute them to epigenetic changes. The epigenetic machinery affected includes DNA methylation, miRNA, and histone modifications (44). The results argue that epigenetic features are critical during neurodevelopment. Any aberration in ongoing epigenetic marks at any stage during neurodevelopment may result in intellectual disabilities. It follows reports that have implicated epigenetic causes in intellectual disabilities (89–91). Such a conclusion has far reaching implications including prospect for an epigenetic therapy (92). We anticipate that this will be a major challenge for the scientific community in the next decade.

We argue that relatively long time course of neurodevelopment offers an opportunity to apply potential epigenetic therapy in intellectual disabilities. For example, a prenatal defect may be corrected following birth during early post-natal development. At this stage, developing brain is malleable. Also, it is responsive to variety of mediators including drugs, care, and social interactions. As stated, it is possible to partially ameliorate FASD deficits by post-natal environmental enrichment in B6 mice. The current most logical mediator for any amelioration in humans appears to be the early environment enrichment including cognitive therapy and interactive schooling. It is considered most logical and effective.

Rodent research also suggests that animals raised under environmentally enriched conditions exhibit relatively fewer stress- and anxiety-like traits. Also, they have improved learning and memory. Further, just like the effect of prenatal alcohol, the recovery of FASD-related cognitive dysfunction due to post-natal environment also involves epigenetic processes. Such results are encouraging for the reversal of epigenetic marks. Although the specific methods for this reversal are not apparent, rehabilitative therapies and drug regimes that target epigenetic pathways would provide a good starting point. To this end, the further research should clarify two aspects of this research. First, what is the relationship between DNA methylation, histone modification and microRNA expression, brain structure and function, and intellectual ability including intellectual deficits in the FASD model? Second, what are the genetic pathways and mechanisms that might be targeted in future attempts to treat behavioral, cognitive, and intellectual deficits associated with human fetal alcohol exposure? The answer to such questions will have the potential to identify suitable treatments for ID caused by neurodevelopmental aberrations.

## Conflict of Interest Statement

The authors declare that the research was conducted in the absence of any commercial or financial relationships that could be construed as a potential conflict of interest.
